# Sling-bridge technique: new technique in extracorporeal septorhinoplasty

**DOI:** 10.3389/fsurg.2024.1369067

**Published:** 2024-04-11

**Authors:** Goran Latif Omer

**Affiliations:** ^1^ College of Medicine, University of Sulaimani, Sulaimani, Iraq; ^2^ Scientific Affairs Department, Smart Health Tower, Sulaimani, Iraq; ^3^ Department of Otorhinolaryngology, Head and Neck Department, Tor Vergata University, Rome, Italy

**Keywords:** extracorporeal septorhinoplasty, sling-bridge technique, facial asymmetry, nasal projection index, septal deviation

## Abstract

**Introduction:**

Extensive septal deviations requiring advanced correction beyond conventional methods. Extracorporeal rhinoplasty technique, involving complete septum removal, reshaping, and reinsertion. While this technique possesses unacceptable risks, the current study aims to introduce a new approach, the sling-bridge technique to enhance and simplify extracorporeal septorhinoplasty, with more tip control and better integrity within the keystone area.

**Methods:**

This prospective analytical study included 50 patients with crooked nasal septum who underwent extracorporeal septorhinoplasty between 2021 and 2023 with the new technique. Patients underwent a full clinical examination, consultation and facial analysis to examine the nose shape and identify any abnormalities and asymmetries in the face.

**Results:**

In the study involving 50 patients, 41 (82%) were males, and with no history of prior rhinoplasty, primarily seeking aesthetic improvements in 44(88%) of participants. Facial asymmetry was observed in 35(70%) of the patients, with 42(84%) individuals exhibiting reduced nasal projection index, nasolabial angle, or nasofrontal angle. The novel technique achieved a straight nose outcome in 45(90%) of patients out of 50, while 5(10%) patients experienced mild deviation linked to extensive preoperative facial asymmetry.

**Conclusion:**

The surgery yielded highly satisfactory results in most patients, with straight nose outcomes being almost 10 times more likely than mild nose deviation outcomes, and no frank deviations. Throughout the follow-up period, none of the patients had complications, especially those that are common in other techniques attributed with extracorporeal septorhinoplasty, such as dorsal irregularities, septal perforation/deviation or abscesses.

## Introduction

One of the most common aesthetic surgical procedures in the world is rhinoplasty, which can be performed by both an otorhinolaryngologist and a plastic surgeon (1). Rhinoplasty is one of the most frequently conducted cosmetic surgeries worldwide, with over 350,000 procedures performed each year in the United States (2). Rhinoplasty can be performed for both aesthetic and functional purposes, and it is very essential for the surgeon to carefully interview the patient to figure out exactly what their main aim and expectation are for the surgery and to thoroughly go through the surgery plan with the patient. This is because patients tend to often raise complaints postoperatively about a matter that was not emphasized or asked for in the preoperative review (3, 4). Since John Roe first detailed the surgery in 1887, it has undergone substantial changes. Since then, the reduction-only technique used for nasal surgery has been gradually replaced by a more proportionate strategy that includes careful reduction and grafting in order to achieve better and more reliable results (5).

Conventional septoplasty and sept rhinoplasty may not suffice for correcting significant nasal septal abnormalities. King, Ashley, and Perret recommended a more radical approach in the 1950s, named extracorporeal rhinoplasty, suggesting complete removal, reshaping, and reinsertion of the septal cartilage for severe deviations (6). Severe septal deviations are usually referred to as a crooked nose and are generally due to birth or childhood trauma. These severe deviations are not always symptomatic, but the more severe the septal deviation, the more likely the patient is to suffer from functional disturbances such as nasal obstructions (7–9). Since Asian patient's tip and the dorsum of the nose are typically in low.

Due to the distinctive ethnic traits commonly found in the noses of Asian individuals, including inadequate and fragile septal cartilage and thick skin, enhancing tip projection can pose challenges (10). The extracorporeal sept rhinoplasty technique presents the surgeon with the opportunity to straighten the septum under direct visualization and also shape the nasal vault, both in a way that results in both proper nasal function and proper nasal form (11). However, it is still considered a procedure with unacceptable risks by several surgeons due to the extensive destabilization of the skeletal structure (specifically around the keystone area) and its technical complexity. In this regard, new surgical techniques have emerged aiming to simplify the procedure and ensure stable fixation of the septum by reattaching the cephalic dorsal neo-septum to the bony-cartilaginous transition zone. These advancements have resulted in a reduced risk of the aforementioned complications. Some examples of these techniques are the criss-cross suture and the transcutaneous trans osseous cerclage suture (12). In the pursuit of a technique with more tip control and better integrity within the keystone area, the current study introduces another technique, which is the sling-bridge technique, that provides a new way of fixing the newly reconstructed neo-septum and upper cartilages to the nasal bones.

## Materials and methods

### Patient population and eligibility criteria

This prospective case series analytical study includes patients who have undergone extracorporeal sept rhinoplasty with the new sling-bridge technique in the Ear, Nose, and Throat departments of Royal Hospital, Sulaymaniyah, between 2021 and 2023. Data such as demographic data (gender, age), affected side, indications for rhinoplasty, previous rhinoplasties was collected.

Patients aged below 18 years old, both genders with straight septum and mildly deviated septum that could be corrected using spreader grafts or other techniques were excluded from this study. Furthermore, patients with normal aesthetic lines and patients who missed any of the assigned follow up visits were also excluded from this study.

The current study includes patients that had a full clinical examination and consultation, through which the nose shape was examined and discussed, also patients who had a crooked nasal septum, which could not be corrected by simple spreader grafts or resection. Patients also were subjected to a complete facial analysis to identify any abnormalities or asymmetry in the face.

The patients were fully informed with regard to all their nasal defects, and the steps needed to get a natural-looking nose were carried out both pre- and intra-operatively. Pre-operative photographs were taken in the clinic studio and included seven different views: front, head up, head down, oblique right, oblique left, left profile, and right profile. The intraoperative photos were taken both following intubation and after the surgery was performed. The otorhinolaryngologist collected all data at a private clinic consensually, and all surgeries were performed by the same specialist. The ethical approval for this study was granted by the ethics committee of the College of Medicine at the University of Sulaimani under Degree No. 12.

### Surgical intervention

**Figure 1:**
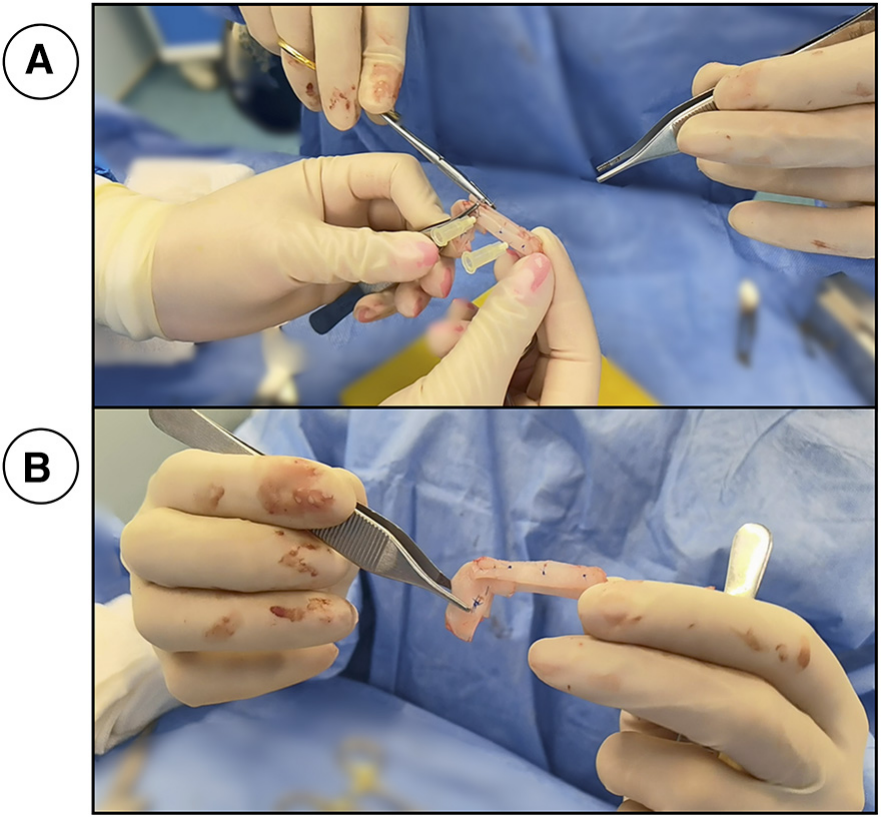
(**A**) shows Two long spreader grafts, an intermediate graft and a columellar graft thar are stitched together to form the neo-septum. (**B**) The final product.

**Figure 2:**
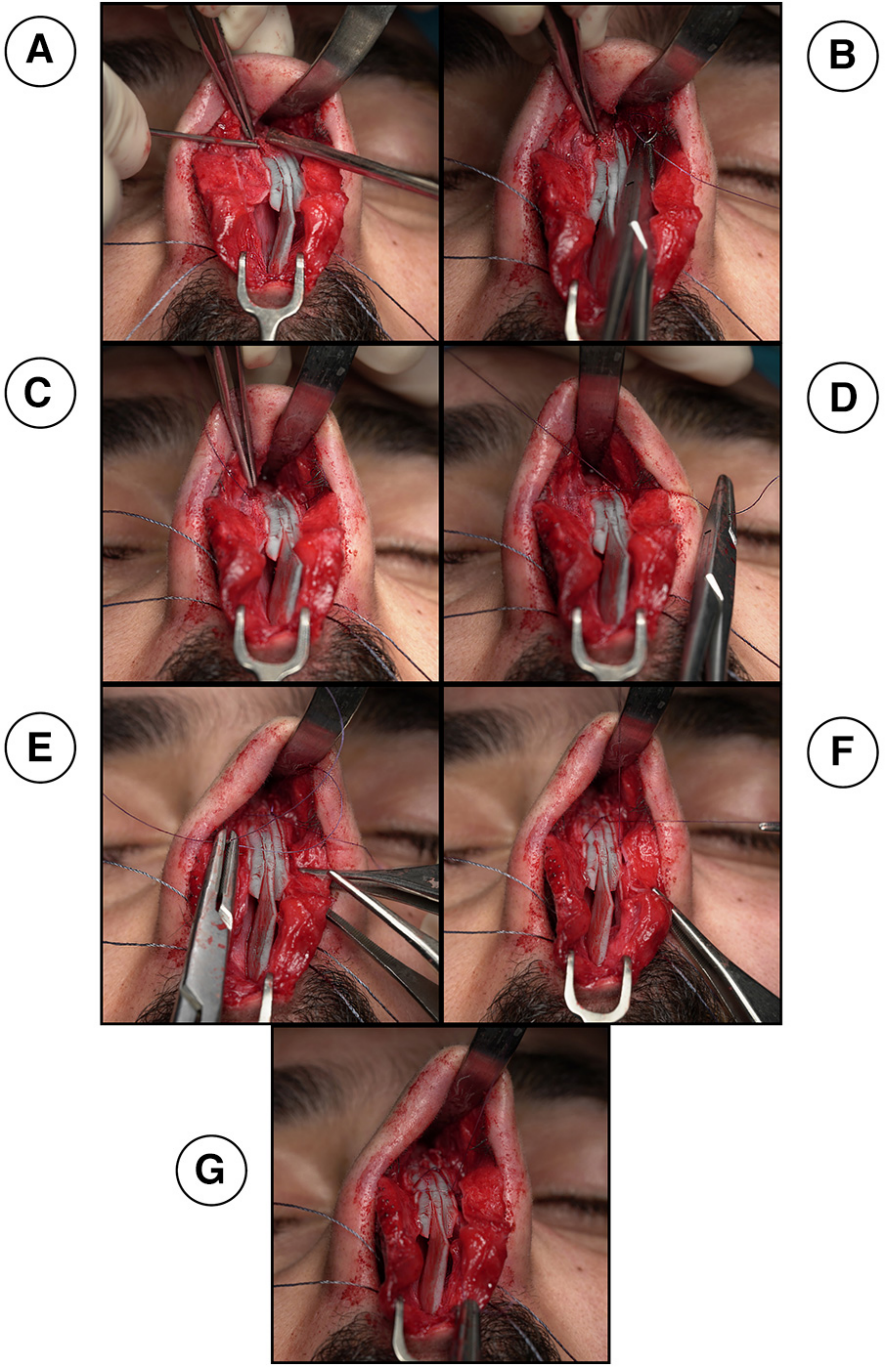
(**A**) creating two holes on each side at the lowest edge of the nasal bone using a pink needle (20G needle). A suture needle goes through the right side of the nasal bone hole (**B**), through the neo-septum and out from the left sided nasal bone hole (**C**) to be tied twice and form a double stitch (**D**) one end of the needle arising from the double stitch crosses over to the opposite side to be inserted into the mid length of the upper lateral cartilage in order to fix the upper lateral cartilage and the neo-septum (**E**) the other end arising crosses over to the other side to also insert into the cartilage at the same level and cross through the same layers so that they can both be tied in the middle portion of the neo-septum (**F**).

Patients were placed under general anesthesia for the surgery in the reverse Trendelenburg position after their appropriateness for anesthesia was evaluated. The operation began with 5.4 ml of lidocaine-epinephrine solution injections into the mucoperichondrial, dorsal skin, nasal floor, and nasal septum. An inverted V-shaped incision, was subsequently made in the columella at its narrowest point, after which the dissection of the lower lateral cartilage, including the lower, middle, and lateral crura, was done. Following those, the dorsum of the nose was dissected, and the superficial musculoaponeurotic system (SMAS) was separated, leading to exposure of the dorsum. Trimming of the lower lateral cartilage was done along with de-humping of the nose using an osteotome, drill, rasp, or chisel, according to the case. Septoplasty was performed using either a partial or complete extracorporeal technique. In cases where there was a partial deviation of the septum, the septum was removed while preserving 1 cm in the upper part of the septum that is attached to the upper lateral cartilage and keystone area. In cases of total deviation of the septum, the entire septum was removed, followed by both medial and lateral osteotomies (internal approach). Moreover, the septum was then cut to create two long spreader grafts: an intermediate graft and a columellar graft, which were then stitched together and placed inside the nose, forming the neo-septum (Figure 1). In cases where there is not enough cartilaginous septum or the quality of the septal cartilage is bad, costal cartilage is harvested. In complete extracorporeal septorhinoplasty, the neo-septum was attached to the uppermost area of the nasal bone by creating two holes on each side at the lowest edge of the nasal bone using either a crusher or a pink needle (20G needle) (Figure 2A). The columellar graft was attached to the two spreader grafts and then to the spine by creating a hole inside the spine using either a drill or crusher, with an intermediate graft in between. The length of the spreader grafts depended on the intraoperative measurement of the nasal bone to the spine, with a height of approximately 5–7 mm and a width of approximately 3 mm. The height of the columellar graft was equal to the distance between the spine and the nasal bone.

**Figure 3:**
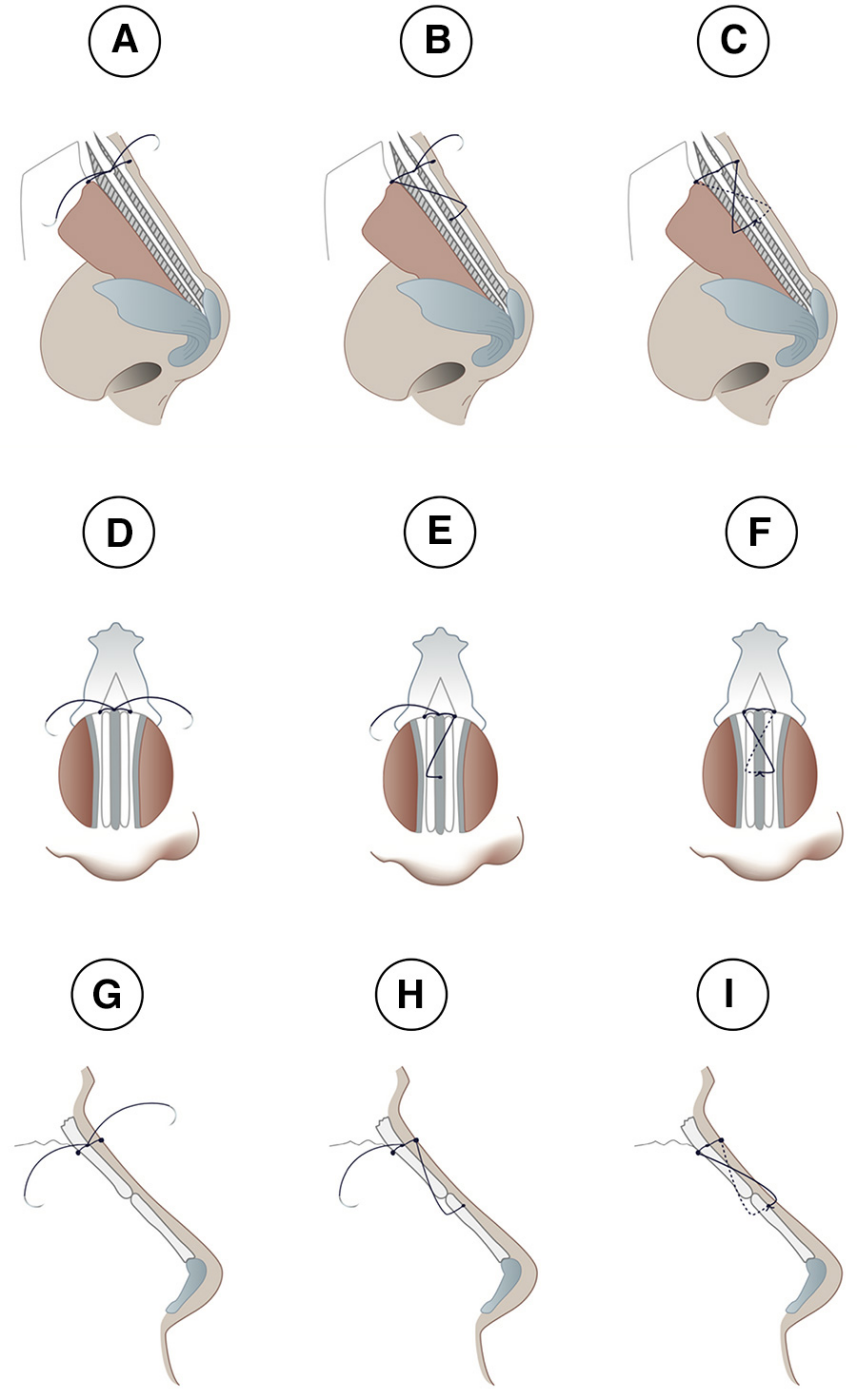
Lateral (**A**–**C** and **G**–**I**) and frontal (**D**–**F**) views of the sling-bridge technique when the dorsum does not require modification. (**A**,**D**,**G**) Show insertion of one end of the needle into the right side of the nasal bone hole and goes through the neo-septum, the same is done with the other end of the suture through the left nasal bone hole, then, both ends are tied twice to form a double stitch. Next, each end of the suture goes through opposing nasal bone holes. In (**B**,**E**,**H**) one end of the needle crosses over to the opposite side to be inserted into the mid length of the upper lateral cartilage in order to fix the upper lateral cartilage and the neo-septum. In (**C**,**F**,**I**), the other end of the needle crosses over to the other side to be inserted into the cartilage also at the same level and cross through the same layers so that they can both be tied in the middle portion of the neo-septum.

**Figure 4:**
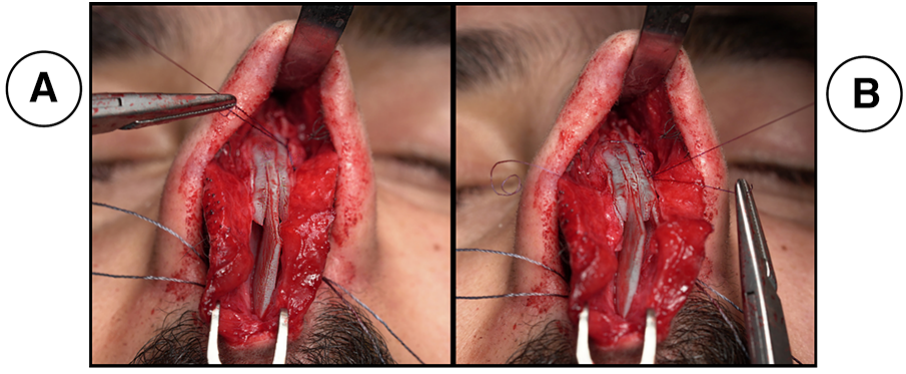
shows cases with dorsum requires further modification, both ends of the needle arising from the double stitch are inserted into the ipsilateral side of the upper cartilage without any crossover occurring over the dorsum (**A**) after insertion, both ends of the needle go through the mid length of the upper lateral cartilage and the neo-septum to be tied (**B**).

**Figure 5:**
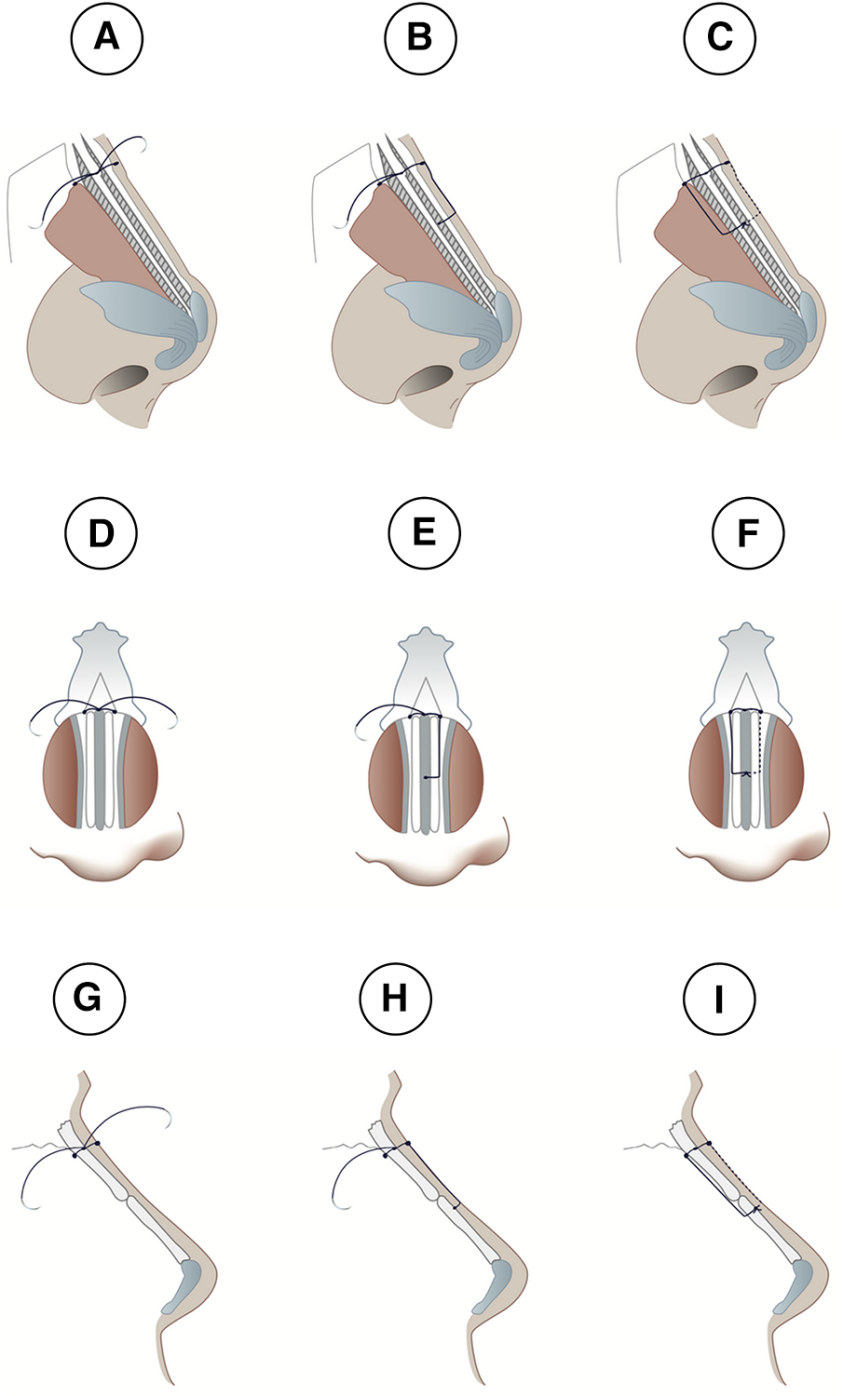
Lateral (**A**–**C** and **G**–**I**) and frontal (**D**–**F**) views of the sling-bridge technique, when the dorsum requires further modification. In (**A**,**D**,**G**), one end of the needle is inserted into the right side of the nasal bone hole and goes through the neo-septum, both ends are tied twice to form a double stitch. In (**B**,**E**,**H**), one end of the needle is inserted into the ipsilateral side of the upper cartilage without any crossover occurring over the dorsum. In (**C**,**F**,**I**), the other end of the needle is inserted into its ipsilateral side of the upper cartilage without any crossover occurring over the dorsum.

**Figure 6:**
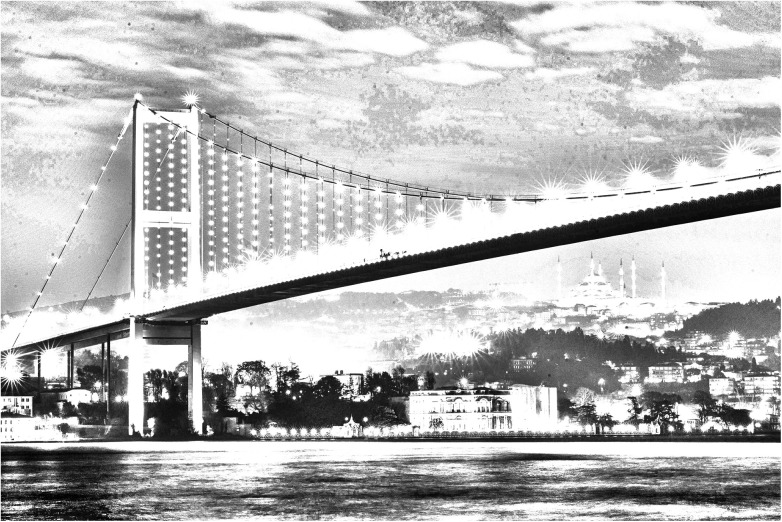
Bosphorus bridge in Istanbul, Türkiye shows the idea behind the sling-bridge technique.

Then, the keystone area is reconstructed between the nasal bone and the neo-septum using the new Sling-Bridge technique. In this new technique, the neo-septum is introduced to the keystone area using a two sided 5.0 PDS suture needle. One end of the needle is inserted into the right side of the nasal bone hole (Figure 2B) and goes through the neo-septum, the same is done with the other end of the suture through the left nasal bone hole (Figure 2C), then, both ends are tied twice to form a double stitch (Figures 2D, 3A,D,G). Next, each end of the suture goes through opposing nasal bone holes. From here on, two different approaches can be followed based on the state of the dorsum and whether or not the surgeon needs to further adjust the dorsum after the sling technique. For cases where the dorsum is straight and requires no further modification, one end of the needle crosses over to the opposite side to be inserted into the mid length of the upper lateral cartilage in order to fix the upper lateral cartilage and the neo-septum (Figures 2E, 3B,E,H). The other end of the needle crosses over to the other side to also insert into the mid length of the cartilage and cross through the same layers so that they can both be tied in the middle portion of the neo-septum to hold the mid portion of the dorsum by the sling force from the nasal bone (Figures 2F, 3C,F,I). For cases where the dorsum requires further modification or when the surgeon is in doubt about it, both ends are inserted into the ipsilateral side of the upper cartilage and neo-septum without any crossover occurring over the dorsum (Figures 4A, 5A,D,G). After insertion, both ends of the needle go through the mid length of the upper lateral cartilage and the neo-septum to be tied similarly to the other approach in the middle portion of the neo-septum (Figures 4B, 5C,F,I). This approach allows the surgeon to work on the dorsum after the sling-bridge technique without the risk of cutting the tie or the stitch. The author resembles this new technique to the Bosphorus bridge in Türkiye (Figure 6), this is because in the Bosphorus bridge, two slings arising from the edge of the bridge are used to hold the middle portion of the bridge. This is similar to the two ends of the needle arising from the double stitch that are inserted into the mid length of the upper lateral cartilage to fix and hold it in order to stabilize all of the dorsum and keep the keystone area still.

Then, tip definition was achieved using tip suturing, VAR (Vertical Alar Resection), and COST (Concomitant Overlap Steal Tip Plasty), all accordingly depending on the shape and length of the lower lateral cartilage. Following that, a symmetrical suture was placed, followed by bilateral crural and cup graft placements. For thick skin or a deprotected nose, a shield graft was used in some cases. The dorsum was then reassessed; tip rotation and projection, nostril modifications, and skin closure were done. Eventually, the septum was quilted.

All of the aforementioned sutures were PDS (Polydioxanone) 5/0. Each patient came back for a follow-up visit in 10 days, 2 weeks, 1 month, 3 months, 6 months, and 1 year following surgery.

### Clinical measures of disease severity

**Figure 7:**
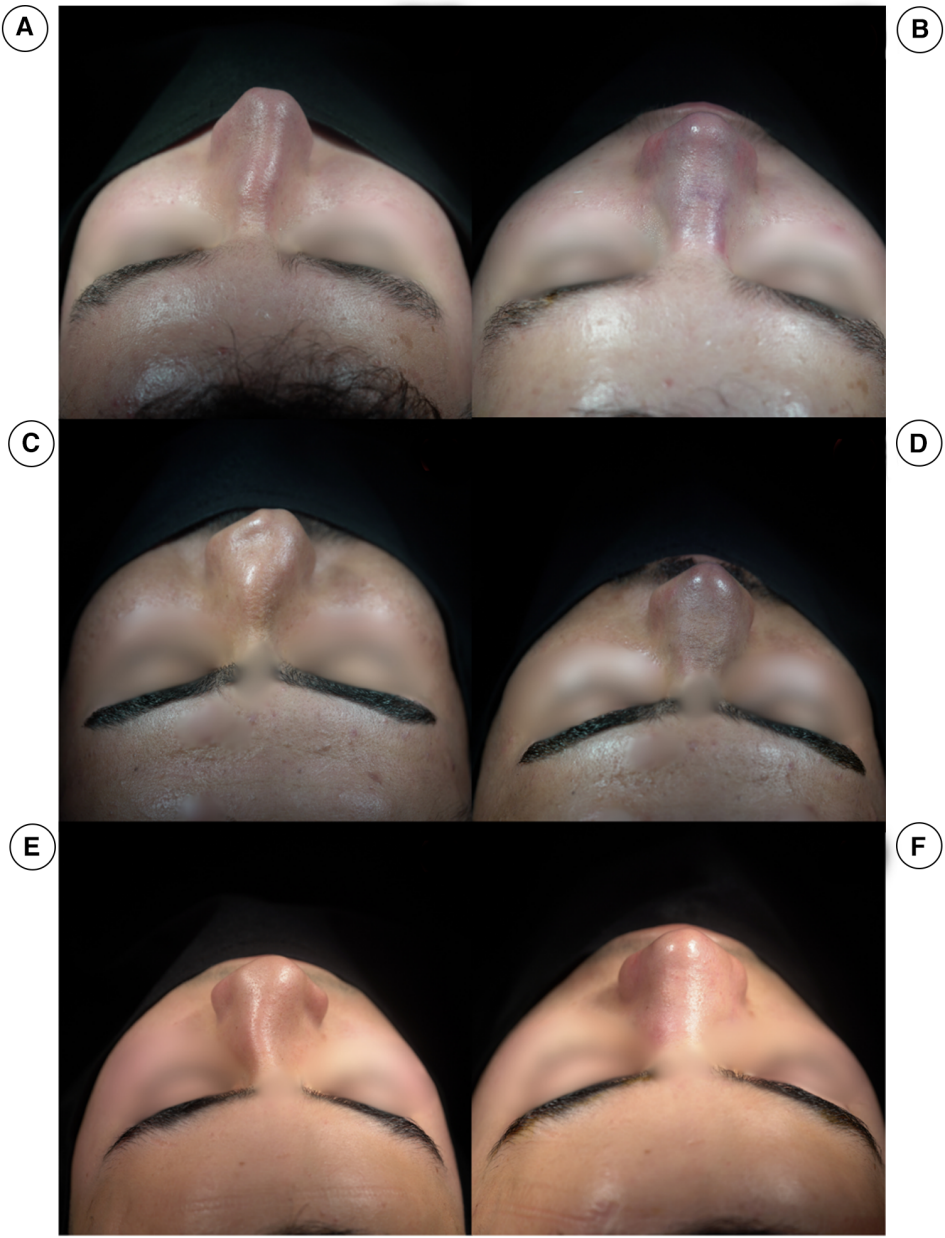
(**A**,**C**,**E**) Show the pre-operation cephalic views of three different patients, all with crooked noses, with the septum towards the right side. (**B**,**D**,**F**) Show post-operation cephalic views of the same patients.

**Figure 8:**
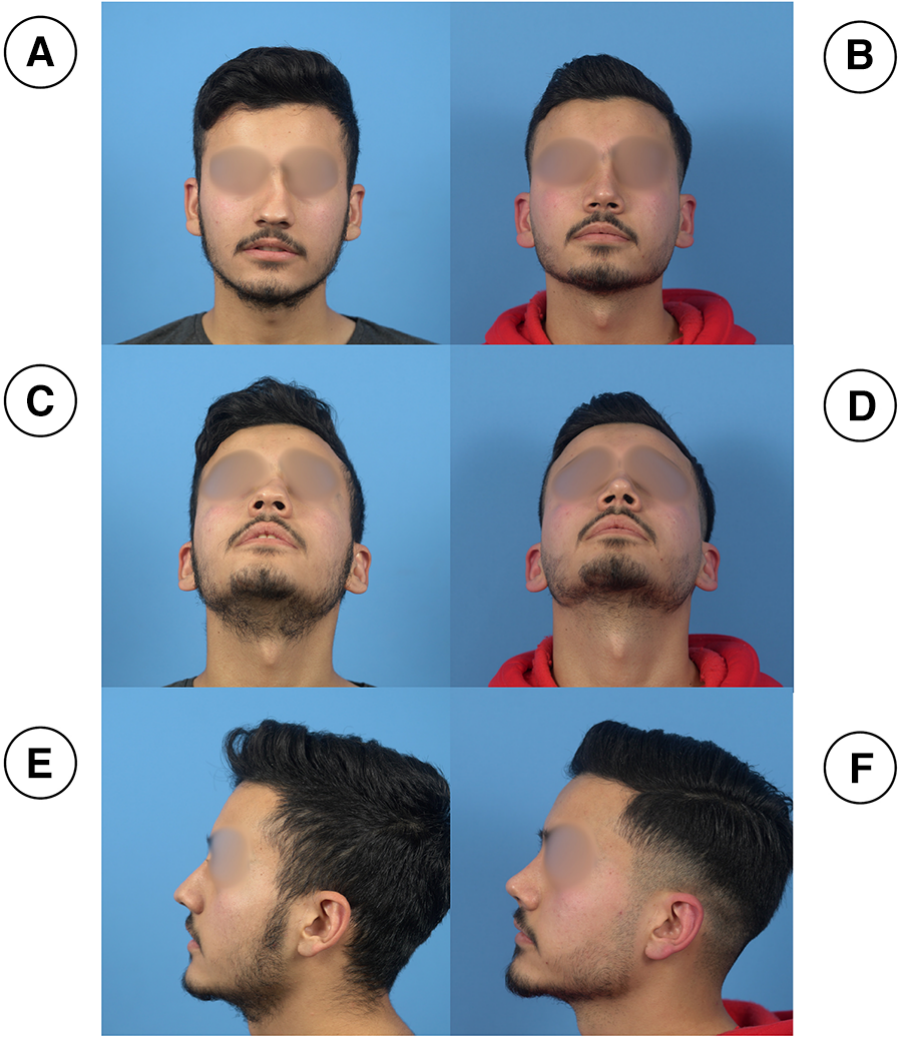
(**A**,**C**,**E**) (preoperative) and (**B**,**D**,**F**) (postoperative, 3 months) photographs of a patient with a crooked nose. The first-row photographs are in frontal view (**A**,**B**), the second-row photographs are in left profile view (**C**, **D**) and the third row photographs are taken in basal views (**E**,**F**).

**Figure 9:**
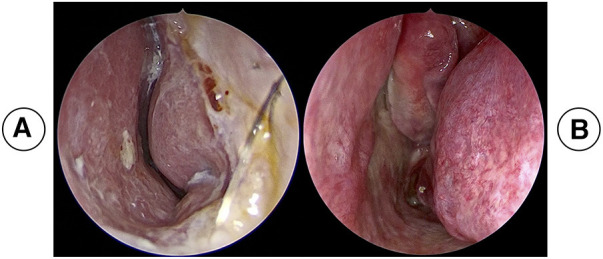
(**A**) (preoperative) and (**B**) (postoperative, 3 months) nasal endoscopic photographs of a patient with a crooked nose.

With regard to the essence of the surgery, the severity of the disease was evaluated through a comprehensive analysis of the medical history pertaining to respiratory complications and thorough clinical examinations. During examination, a meticulous facial analysis was done, which included assessing the ratio between nasal length and tip projection (about 0.67), checking the nasofrontal (115°–130°), nasolabial (90°–95° for males and 95–115° for females), nasofacial (30°–40°), and nasomental angles (142°–152°), the alar base width (31–33 mm), radix analysis, and examining the quality of skin. Additionally, the aesthetic outcomes were further scrutinized and recorded through perioperative documentation, as all patients were photographed preoperatively, instantly after the operation (Figure 7), and throughout the follow-up period (10 days, 2 weeks, 1 month, 3 months, 6 months, 1 year). Throughout the follow up period, none of the patients had dorsal irregularities, saddle nose, septal perforation/deviation or abscesses (Figures 8 and 9).

### Primary outcomes of the current study

The primary outcome of the study is that the crooked nose and septum is fixed and optimal aesthetic lines and angles are achieved. Furthermore, this technique seeks to enhance nasal function by opening both the internal and external nasal valves and correcting the septum.

### Data administration and statistical analysis

Data entry was done using Microsoft Excel (For Mac) Version 16.78.3, then, all of the data were imported into IBM SPSS Version 26.0.0.0. Descriptive statistics such as frequency and percentage were found for the variables.

## Results

Of the total patients enrolled in this study (*n* = 50), 41 (82%) were males and 9 (18%) were females. Patients mean age was 28 years old (ranging between 18 and 54 years old). Furthermore, young patients seemed to be the most common age group, with the 18–24 and 25–34 age groups together making up close to 74% of the participants. operations were carried out from 2021 to 2023. Among all patients undergoing rhinoplasty, 44(88%) of them underwent rhinoplasty for the first time. In terms of the affected side of the nose, 24(48%) of individuals had only a left-sided issue. All patients were followed up at 10 days, 2 week, 1 month, 3 month, 6 month and lastly, at 1 year.

Regarding surgery indications, among all patients, 40(80%) of them cited both aesthetic and functional reasons. Among those with aesthetic concerns, 21(42%) reported severe deflection of both caudal and dorsal septa, deformities in nasal bony structures, and a twisted nose. Conversely, 43(86%) individuals with functional concerns primarily involve their internal nasal valves.

Facial symmetry was prevalent in 35(70%) of individuals, with 40(80%) exhibiting decreased values for the nasal projection index, nasolabial angle, and nasofrontal angle. Regarding the outcome, 45(90%) had straight nose.

Following intraoperative evaluations, 38(76%) patients were selected for septal cartilage graft. The most common reduction method was de-humping by chisel observed in almost 20(40%) of the patients. half of the patients, a portion of the bony septum was remaining following the surgery. Postoperative supratip depression was observed in only 1 (2%) of the patients whereas none of the patients had postoperative inverted V deformity.

Additional details regarding the characteristics of the participants included in the study can be found in Table 1.

**Table 1:** Demographic and clinical characteristics of participants enrolled.

Variables	Frequency	Percentage (%)
Gender		
Male	41	82
Female	9	18
Age		
18–24	22	44
25–34	15	30
35–44	12	24
45–54	1	2
Previous rhinoplasties		
1 Previous rhinoplasty	4	8
2 Previous rhinoplasties	2	4
None	44	88
Affected side		
Right side	15	30
Left side	24	48
Both sides	11	22
Indication for rhinoplasty		
Aesthetic	7	14
Functional	3	6
Both	40	80
Causes of aesthetic indication		
Severe deflection of the caudal septum	6	12
Severe deflection of the dorsal septum	8	16
Deformities of the nasal bony structures	6	12
Twisted nose	6	12
All of the above	21	42
Not aesthetic	3	6
Functional/internal nasal valve involved?		
Yes	43	86
No	7	14
Nasal projection index decreased?		
Yes	39	78
No	11	22
Nasolabial angle decreased?		
Yes	42	84
No	8	16
Nasofrontal angle decreased?		
Yes	39	78
No	11	22
Facial symmetry		
Yes	35	70
No	15	30
Type of skin		
Thick skin	11	22
Thin skin	19	38
Normal skin	20	40
Skin quality		
Normal	34	68
Elastic Rigid	11	22
	5	10
Outcome		
Straight nose	45	90
Mild deviation	5	10
Types of septal cartilage graft		
Costal graft	6	12
Auricular graft	6	12
Septal	38	76
Reduction method		
Rasping	10	20
De-humping by chisel	20	40
Drill	12	24
No reduction	8	16
Deviation of the septum at the keystone area		
Yes	32	64
No	18	36
Level of septum deviation		
High deviation	40	80
Low deviation	10	20
Site of deviation within the septum		
Bony part deviation	1	2
Cartilaginous part	15	30
Both	34	68
Nasal bone length		
Short	15	30
Long	35	70
Dislocated nasal spine		
Yes	37	74
No	13	26
Type of fixation to the nasal spine		
Direct fixation	39	78
Fixation with spacer graft	11	22
Is there a part of the bony septum remaining?		
Yes	26	52
No	24	48
Inverted V deformity		
Yes	0	0
No	50	100
Postoperative supra tip depression		
Yes	1	2
No	49	98

## Discussion

Correcting severe nasal deviations has been a challenge for many surgeons because classical septorhinoplasty techniques in these cases do not have acceptable outcomes and end up with either unfavourable results or relapse (13). That is how the concept of extracorporeal septorhinoplasty was born. In the literature, it is shown that, apart from the appealing aesthetic results, the extracorporeal septorhinoplasty technique has also shown better postoperative functional results when compared to the *in situ* septal correction techniques in rhinoplasty (14).

Additionally, in a study by Wilson and Mobley, it was observed that the rates of complications for extracorporeal septoplasty are comparable to those observed in endonasal septoplasty (15).

However, even though this approach has resolved many issues, it comes with its own drawbacks. First, extracorporeal septorhinoplasty has many technical difficulties, which include end block separation of the original septal cartilage from the anterior nasal spine and keystone areas and the achievement of secure fixation of the neo-septum to the anterior nasal spine and keystone area, both of which challenge even for highly experienced surgeons (10). Moreover, there are many minor details in the surgery that can cause devastating results; for example, overly tight quilting sutures and aggressive packing post-operatively can cause necrosis of the intervening tissue, thus separating the neo-septum from its designated base (16). Next, extracorporeal rhinoplasty has many complications, most notably dorsal irregularity (i.e., saddling of the dorsum or irregular dorsal contour). In addition, it can also cause renewed septal deviations, septal perforation (which is due to tearing or devascularization of the mucoperichondrial flaps), stiffness of the upper lip, and abscesses (6, 16).

Due to these complications and technical difficulties, many surgeons have hesitated to do this surgery, while encouraging researchers to find different ways to modify the surgery so as to make it more efficient. One of the techniques is using a criss-cross suture (12).

Another technique is the transcutaneous transosseous cerclage suture, which is used when the nasal bones are not long enough for a criss-cross suture (12).

However, in this study, another technique is presented, called the sling bridge technique. The aim of developing this technique was to lessen the trauma on the nasal bones, since the extensive bone destruction that is done in other techniques increases the chance of dorsal irregularities and weakens the overall framework of the nose. Also, another important benefit of this new technique when compared to the others is that it has more anterior dorsal support and is thus less likely to cause a saddle nose deformity in the long run. In the current study, encompassing a total of 50 surgical cases, it was observed that 90% of the procedures yielded a straight nose, while the remaining 10% exhibited only mild deviations. The latter instances were attributed to pre-existing severe facial asymmetries in the respective cases.

This study had its own set of limitations, primarily the restricted number of participants involved. Consequently, we strongly advocate for the implementation of this technique in future multi-centered studies, alongside a comprehensive comparison with alternative techniques.

The current study encountered certain limitations, most notably the limited number of participants. Consequently, we strongly advocate the incorporation of this technique into forthcoming multicenter studies, coupled with a thorough examination through comprehensive comparisons with alternative techniques.

## Conclusion

Conventional septoplasty and septorhinoplasty may not be sufficient for correcting significant nasal septal abnormalities. As a result, a more radical approach called extracorporeal septoplasty has emerged. While new surgical techniques have been developed to simplify the procedure and ensure stable fixation of the septum, none of them are without complications. In our pursuit of a technique that lessens the drawbacks of this procedure, we have introduced a new technique known as the sling-bridge technique. This new surgical technique yielded highly satisfying results for all of the patients in the study. Throughout the follow-up period, none of our patients have experienced complications such as dorsal irregularities, septal perforation or deviation, or abscesses.

## Data Availability

The original contributions presented in the study are included in the article/Supplementary Material, further inquiries can be directed to the corresponding author.
